# The effect of environmental variation on stable coexistence of competitors: experimental evidence from zooplankton (*Daphnia magna* and *D. pulex*)

**DOI:** 10.1093/plankt/fbag019

**Published:** 2026-03-28

**Authors:** Sigurd Einum, Tim Burton, Silje M Larsen, Varsha Rani, Aline M Lee

**Affiliations:** Centre for Biodiversity Dynamics, Department of Biology, Norwegian University of Science and Technology, NO 7491 Trondheim, Norway; Centre for Biodiversity Dynamics, Department of Biology, Norwegian University of Science and Technology, NO 7491 Trondheim, Norway; Norwegian Institute for Nature Research, NO 7491 Trondheim, Norway; Centre for Biodiversity Dynamics, Department of Biology, Norwegian University of Science and Technology, NO 7491 Trondheim, Norway; Norwegian Institute for Nature Research, NO 7491 Trondheim, Norway; Centre for Biodiversity Dynamics, Department of Biology, Norwegian University of Science and Technology, NO 7491 Trondheim, Norway; Institute of Aquatic Ecology, HUN-REN Centre for Ecological Research, H 1113 Budapest, Hungary; Doctoral School of Biology, Institute of Biology, ELTE, Eötvös Loránd University, H 1117 Budapest, Hungary; Centre for Biodiversity Dynamics, Department of Biology, Norwegian University of Science and Technology, NO 7491 Trondheim, Norway; Gjærevoll Centre for Biodiversity Foresight Analyses, Norwegian University of Science and Technology, NO 7491 Trondheim, Norway

**Keywords:** species coexistence, environmental variation, environmental fluctuations, population, dynamics, competition, niche, temperature, zooplankton

## Abstract

Coexistence of competing species may be influenced by environmental variation. Specifically, theory suggests that short-term environmental variability can contribute to long-term coexistence among competitors. Here, we address the role of environmental variation on competitive interactions between two zooplankton species (*Daphnia magna* and *D. pulex*) which are found sympatrically, but where mechanisms allowing for such coexistence remain unclear. Using competition experiments, we show that under constant temperature conditions, one of the species (*D. magna*) was greatly outnumbered by their competitor (*D. pulex*). Furthermore, population simulations showed a significant possibility for extinction of the inferior competitor, and distributions of estimated niche differences and relative fitness differences included parameter sets that precluded stable coexistence. Under fluctuating temperature conditions, however, the numerical dominance by *D. pulex* was considerably reduced. Moreover, under these conditions the occurrence of extinction of *D. magna* in the simulations became negligible, and all parameter sets drawn from the estimated distributions of niche differences and relative fitness differences met the requirements for stable coexistence. Our results provide empirical support for previous model results showing how short-term variation in temperature can promote species coexistence.

## INTRODUCTION

Environmental variation is a key factor impacting biodiversity. According to the intermediate disturbance hypothesis (Connell, 1978), high levels of environmental variation (frequency or magnitude of "disturbance") lead to low diversity of competing species because few of them are able to colonize and reproduce when conditions are favourable. Low levels of environmental variation also lead to low diversity because species exclusion by superior competitors will occur. Maximum diversity is then maintained at intermediate levels of variation where these processes have less influence.

Whilst the above deals with non-equilibrium situations, where coexistence is only temporary, the relative abundance of different competing species under stable coexistence, and ultimately their potential for such coexistence, also depends on environmental variation. Coexistence theory states that the outcome of competition is driven by the degree of niche overlap between competitors (determining how much they compete) and the fitness differences between them if they were to utilize exactly the same niche (Chesson, 2000). Both theory and empirical data suggest that stable coexistence is facilitated by niche segregation (i.e., less niche overlap), causing intraspecific competition to be greater than interspecific competition (Adler *et al.,* 2018; Chesson, 2000). Thus, if environmental conditions influence competition, this will alter the potential for coexistence in an indirect way, additional to the direct effects of the environment on individual species' fitness.

Mounting evidence suggests that changes in community composition following changes in the environment cannot be predicted based on direct single-species responses to environmental conditions, because community responses will be shaped by such environmental effects on competitive interactions (e.g. Davis *et al.,* 1998; Hart and Marshall, 2013; Milazzo *et al.,* 2013; Stenseth *et al.,* 2015; Armitage and Jones, 2019). In most cases, such studies have focused on the effect of mean environmental conditions (but see Armitage and Jones 2019). However, as for coexistence under non-equilibrium situations, stable coexistence of competing species may also be influenced by environmental variance.

Chesson (1994) showed that short term environmental variability can contribute to long-term coexistence among competitors. This prediction is supported by empirical observations within different types of communities (prairie grasses [Adler *et al.,* 2006], annual plants [Pake and Venable 1995; Facelli *et al.,* 2005; Angert *et al.,* 2009], rodents [Brown, 1989]. However, perhaps the largest proportion of studies within this field stems from aquatic phytoplankton communities. Phytoplankton communities have historically received considerable interest regarding the mechanisms maintaining species diversity. In his paper "The paradox of the plankton", Hutchinson (1961) pointed out the discrepancy between Gausse's competitive exclusion principle and the observation that lakes typically harbor a large number of phytoplankton species that compete for the same resources, that have little opportunity for microhabitat segregation, and that thus presumably have extensive niche overlaps. Although a diverse set of explanations for the maintenance of phytoplankton diversity has been proposed (Roy and Chattopadhyay, 2007), several experimental studies point to temporal environmental variation (nutrients, light, temperature) in playing a role for preventing competitive exclusion and facilitating species coexistence in these communities (e.g. Robinson and Sandgren, 1983; Sommer, 1985; Spiejkerman and Coesel, 1996; Flöder *et al.,* 2002; Descamps-Julien and Gonzalez, 2005). Similar observations have been made in experiments on freshwater ciliated protozoa (Eddison and Ollason, 1978; Jiang and Morin, 2007).

The observations in phytoplankton and protozoa outlined above suggest a role of environmental variation in aquatic plankton communities in general. However, in comparison to the substantial amount of work that has been done on phytoplankton, much less effort has been made to understand the role of environmental variation on communities of multicellular zooplankton. Particularly within the filter feeding component of these communities (e.g. cladocera) it commonly consists of species that to a large extent compete for a similar diet of phytoplankton and bacteria, and thus presumably have greatly overlapping niches (Ghilarov, 1984). Such a "paradoxical" situation extends to the maintenance of multiple obligate parthenogenetic clones of a single species within single communities (Hebert and Crease, 1980). Correlative evidence suggests that environmental variation has a role in maintaining this variation. Specifically, among a set of 53 lakes, higher zooplankton richness was found in lakes that showed greater temperature variation at different temporal scales (interannual, seasonal and residual, Shurin *et al.,* 2010). Furthermore, in a long-term study of a single lake, Cáceres (1997) found that among two competing species of zooplankton (*Daphnia galatea mendotae* and *D. pulicaria*), only one would be sustained long-term in the absence of environmental inter-annual variation. One potential mechanism for such effects of temperature fluctuations could be that thermal performance curves vary among species, and even among clones within species (Fossen *et al.,* 2018). Modelling suggests that, in combination with short-term variation in temperature, this may promote species coexistence by reducing the average fitness difference between species (Liu *et al.,* 2021).

In the current study we test how environmental variation impacts competitive interactions between two cladoceran zooplankton species, *D. magna* and *D. pulex*. Despite their similarities in morphology (Ranta *et al.,* 1993) and niche characteristics (Pajunen and Pajunen, 2007), they are found sympatrically in nature (Hanski and Ranta, 1983) and may coexist for multiple years in artificial pools (Bengtsson, 1993). Experimental work indicates that their competitive relationships may depend on mean temperatures (Bengtsson, 1986), but it is not known how this might be affected by the more ecologically realistic scenario where temperature can fluctuate. Temperature is an environmental variable that has a fundamental effect on organismal biology, particularly for ectotherms, and that varies greatly at different temporal scales. Thus, we focus on temperature as our environmental variable, and conduct experiments under different levels of temperature variation. Regular censusing of populations allows us to quantify intra- and inter-specific competition coefficients, to compare these among temperature variation regimes, and to address the question of whether the relative abundance and/or the potential for coexistence of these two species is influenced by temperature variation.

## MATERIALS AND METHODS

Stock cultures consisted of a single clone of *D. magna* from northern Norway (Sandtjønna, 67.687°N 12.672°E) and a single clone of *D. pulex* from central Norway (Lake Asklundvatnet, 63.588°N, 10.729°E). Both clones had been reared in the lab at 17 °C for multiple generations. For logistical reasons, experimental populations were established at two times (hereafter referred to as blocks), one on the 23^rd^ – 24^th^ February, and one on the 9^th^ – 10^th^ March 2021. The populations were initiated by transferring three neonates (< 48h) of each species into each of forty-six 600ml glass jars filled with 500ml of ADaM (medium suitable for rearing *Daphnia*, Klüttgen *et al.,* 1994). To standardize the starting conditions in terms of microbiota communities, all jars were inoculated with 2.5ml of filtered (1.2 μm) medium from each of the two stock culture types. During the first week following initiation each received 0.0375 ml of Shellfish Diet 1800 (Reed Mariculture Inc.), corresponding to a final algae concentration of 1.5 × 10^5^ ml^-1^, every 2-3 days. Eight days after initiation they were culled, leaving two individuals from each species, which served to standardize starting densities in case of mortality during the initial establishment period (no reproduction had occurred at this time). Thereafter, 0.1125 ml of Shellfish Diet, corresponding to a final algae concentration of 4.5 × 10^5^ ml^-1^, was added three times per week for the remainder of the experiment. This concentration was chosen to ensure ad lib food abundance during population establishment, and subsequent food limitation as populations grew (Fossen *et al.,* 2021).

To further ensure identical starting points of the experimental populations, they were kept at 17 °C until one week after the culling, which is when temperature treatments were initiated. Populations were assigned to one of three treatments: (1) constant at 17 °C (C, N = 7 and 8 populations in first and second block, respectively), (2) a low amplitude fluctuation (LF, N = 8 and 8 populations in first and second block, respectively) consisting of a weekly shift between 14 and 20 °C, and (3) a high amplitude fluctuation (HF, N = 7 and 8 populations in first and second block, respectively) consisting of a weekly shift between 10 and 24 °C. The age at maturation for these species decreases with increasing temperature and typically ranges between ~7 and 15 days at the temperatures used here when fed *ad lib* (Fossen *et al.,* 2018; Van Geest *et al.,* 2010). Thus, in the fluctuating treatments, most individuals should experience at least one shift in temperature prior to first reproduction. The temperature range was chosen to ensure positive population growth for both species (Van As *et al.,* 1980; Adamczuk, 2020). Temperature fluctuation was implemented by moving the jars between different climate cabinets that were set at the different temperatures. For both the HF and the LF treatments, the first temperature shift was a move to a higher temperature. For all temperature treatments a 16L:8D light regime was provided throughout the experiment.

Sampling of populations started four days after the second temperature shift (26 days after initiation). By waiting with the sampling until this time, we ensured that all individuals from the initiation had started reproducing and most likely were grandmothers. Furthermore, this ensured that any effect of treatments had started to manifest. Sampling was performed weekly until 3^rd^ May, such that the experiment lasted for 69 and 55 days for blocks 1 and 2, respectively. These durations appeared sufficient to allow the populations to go into fluctuations around the carrying capacity (Fig. S1). This resulted in seven population censuses in the first block, and five censuses in the second block. Populations were sampled by stirring the content of each jar to homogenize the distribution of animals and then siphoning out 100 ml of medium for filtration and ethanol fixation. Removed volumes were replaced with an equal amount of ADaM. In addition to the medium change occurring during sampling, additional medium changes (complete replacement) and detritus removal from the bottom of jars was conducted every 2 weeks.

All sampled individuals were identified to species in samples containing ≤ 50 individuals. For larger samples we identified the first 50 individuals encountered when the sample was distributed along a counting tray and counted the remaining ones while assuming an equal representation of the two species in the latter. These data were used to calculate the total population size for each species at each sampling event in each population (Fig. S1). Population sizes were used to calculate species-specific daily population growth rates, *G*, between each consecutive pair of samples at times t-1 and t as ln(population size prior to sampling_t_ /population size after sampling_t-1_)/(number of days). Samples that did not contain one of the species could not be used for calculation of growth rate for that species over the last interval leading up to this census. The growth rate over the interval of that species was then set to NA. If a species was absent in one sample but reappeared in later samples, the growth rate was calculated over this longer time period (e.g. if a species was lacking from census 4, but detected in census 3 and 5, growth rate was calculated over this latter interval).

## STATISTICAL ANALYSES

All statistical analyses were conducted in R v.4.2.3 (R Development Core Team, 2023). To model the temporal variation in abundance throughout the experiment, linear mixed-effect models were fitted using the function *lme* in the package *nlme* (Pinheiro *et al.,*, 2021). The full models contained the fixed main effects of block, sampling event (as factor), species ID and treatment. In addition, we included an interaction term between species ID and sampling event, and one between species ID and treatment. Population ID was included as a random intercept.

Population dynamics parameters were estimated using the Lotka-Volterra model


(1) \begin{equation*} {G}_i={r}_i\left(1-{\left({\alpha}_{ii}{N}_i+{\alpha}_{ij}{N}_j\right)}^{{c}_i}\right), \end{equation*}

where subscripts indicate species identity, ${\alpha}_{ii}$ is a measure of the strength of intraspecific competition and ${\alpha}_{ij}$ of interspecific competition (effect of species *j* on species *i*). *G* is the observed daily growth rate between two censuses (see above), *r* is the intrinsic population growth rate, and *N* is population abundance. The constant *c* allows for a non-linear relationship between growth rate and total daphnia abundance. In our results, *i* refers to *D. magna* and *j* to *D. pulex*. Different versions of the model were fitted using the function *nlme* in the package *nlme* (Pinheiro *et al.,* 2021), either including or excluding temperature treatment effects on intra- and interspecific competition coefficients and intrinsic population growth rate. All models contained a random effect of population on *r*. Different models were compared using AICc.

Based on the best fitted model, we used estimates of competition coefficients to calculate niche differences, $\mathrm{ND}=1-\sqrt{\frac{\alpha_{ij}{\alpha}_{ji}}{\alpha_{ii}{\alpha}_{jj}}}$, and relative fitness differences, $\mathrm{RFD}=\sqrt{\frac{\alpha_{ij}{\alpha}_{ii}}{\alpha_{jj}{\alpha}_{ji}}}$, and evaluate whether the conditions for coexistence,


(2) \begin{equation*} {\displaystyle \begin{array}{c}1-\mathrm{ND}<\mathrm{RFD}<\frac{1}{1-\mathrm{ND}},\end{array}} \end{equation*}

are met in these two species (Carroll *et al.,* 2011; Chesson, 2018; Godwin, *et al.,* 2020). The first term, $1-\mathrm{ND}$, is a measure of niche overlap between the two species. If this term equals zero, the species have no shared resources, whereas if it is one, they have identical niches. RFD is a measure of the relative fitness of the two species. When the inequality in (2) is met, the niche overlap is small enough to prevent competitive exclusion of the species with lower fitness. This might cause greater uncertainty in the parameter estimates, since we are estimating all parameters of the Lotka-Volterra model in equation (1) in a single model-fitting step which provides the estimation procedure with less information for partitioning variation into contributions from intra- and interspecific drivers. It is therefore critical to consider this uncertainty (as described below) when drawing conclusions. Additionally, while the original method described by Godwin *et al.* (2020) assumed linear density dependence, they showed that when density dependence is non-linear, as in our system, estimates of $\frac{1}{K_i}$, where ${K}_i$ is carrying capacity of species *i*, give more accurate results than intraspecific coefficients measured from a linear regression near equilibrium in a monoculture. In the model used here (equation (1)) ${\alpha}_{ii}$ is equivalent to $\frac{1}{K_i}$. At equilibrium, equation (1) becomes the same as a linear model, and our estimates of the interspecific competition coefficients are therefore equivalent to those expected from a linear regression near equilibrium, as used by Godwin *et al.* (2020). We evaluated whether the conditions in equation (2) were met in each temperature treatment based on ND and RFD. Values of ND and RFD were first calculated directly from the estimated competition coefficients. In addition, to account for the uncertainty in the estimates, we drew 500 000 sets of coefficients from the estimated distributions and calculated how many of these led to conditions for coexistence being met.

To predict equilibrium population abundances for the two species when in competition we used the parameter estimates from the best model to simulate population dynamics according to equation (1). Simulated populations were initiated with one individual of each species and were allowed to grow with daily increments until reaching equilibrium using the Lotka-Volterra model. For each level of temperature fluctuation, model parameters were drawn from a normal distribution based on the corresponding estimated mean and SE in that environment, and 100 000 simulations were conducted.

## RESULTS

### Population abundance

**Fig. 1:**
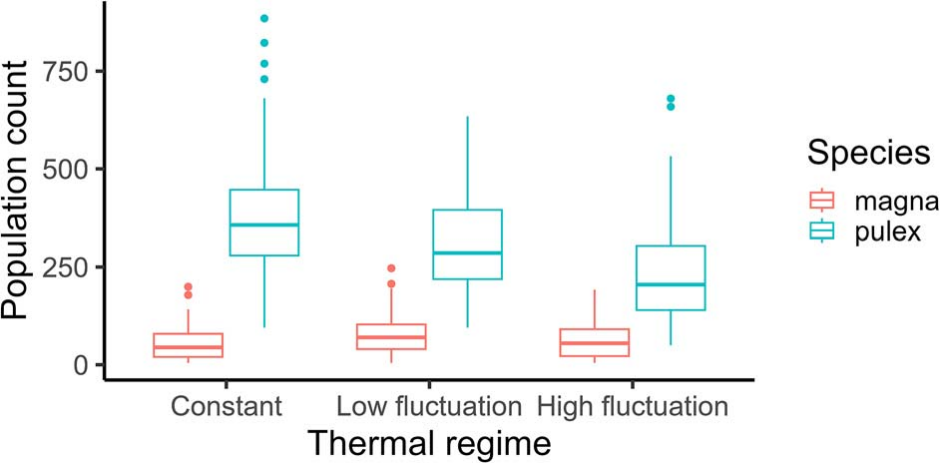
Population abundance of *Daphnia magna* and *D. pulex* when in competition under the three temperature regimes; constant (C), low fluctuations (LF) and high fluctuations (HF). Data from seven censuses are pooled (for temporal patterns see Figs. S1, S2).

*D. pulex* generally maintained higher population abundance than *D. magna* throughout the experiment (Fig. S2). However, the magnitude of this difference depended on the thermal regime, as the best model contained a species by temperature treatment interaction (Table S1). This was caused by a decline in abundance with increasing temperature fluctuations for *D. pulex*, which was absent for *D. magna* (Fig. 1).

### Population growth rates

For *D. magna*, model comparisons suggested that both the strength of competition from *D. pulex* and the intrinsic growth rate (*r*) was independent of treatment (Table S2). Furthermore, there was little support for a treatment effect on the strength of intraspecific competition (Table S2). For *D. pulex*, there was strong evidence for a treatment effect on the strength of intraspecific competition, and somewhat weaker support for a treatment effect on the strength of competition from *D. magna* as well as for a treatment effect on the intrinsic population growth rate (Table S2).

Inspection of parameter values from the best model (Table 1) reveals that for *D. magna*, the intraspecific competition coefficient was 4.5 times as large as the interspecific one and was independent of temperature treatment. In contrast, for *D. pulex* the interspecific coefficient was 2.2 times that of the intraspecific one under constant temperature. However, as temperature variability increases, their interspecific coefficients decrease and intraspecific coefficients increase, such that the relative magnitude of these two are reversed at the high level of temperature fluctuations (Table 1). In addition, *r* decreased with increasing temperature fluctuation for *D. pulex* (Table 1).

**Table 1:** Parameter estimates ± SE from the best-fitting models analyzing variation in population growth rate (ΔAICc = 0, Table S2) fitted to data from competition experiments between *D. magna* and *D. pulex* that were performed under different patterns of temperature variation. Parameters are presented according to the presence/absence of interactions with temperature treatment. Thus, population dynamic parameters that did not depend on the pattern of temperature variation are presented under the heading ‘Common’, whereas parameters that were found to depend on the pattern of temperature variation are shown separately for each of the temperature treatments.

	Common	Constant	Low fluctuation	High fluctuation
*D. magna*				
*a_ii_*	0.0063 ± 0.0008	–	–	–
*a_ij_*	0.0014 ± 0.0002	–	–	–
*r*	0.1170 ± 0.0144	–	–	–
*c*	1.5885 ± 0.3144	–	–	–
*D. pulex*				
*a_jj_*	–	0.0017 ± 0.0001	0.0019 ± 0.0002	0.0027 ± 0.0002
*a_ji_*	–	0.0037 ± 0.0008	0.0016 ± 0.0007	0.0012 ± 0.0009
*r*	–	0.1860 ± 0.0130	0.1627 ± 0.0119	0.1458 ± 0.0120
*c*	0.8697 ± 0.1011			

### Conditions for coexistence

Conditions for coexistence (equation 2) were met in all three temperature treatments when calculated based on estimated parameters without accounting for uncertainty (S: 0.69 < 1.17 < 1.45, LF: 0.43 < 1.69 < 2.33, HF: 0.31 < 1.64 < 3.25). This potential for coexistence is mirrored by the observed abundances of the two species in the final census (Fig. S2). We see that the estimate of niche overlap decreases from 0.69 to 0.31 as the temperature becomes more variable. When parameter estimates were drawn from the estimated distributions in Table 1, conditions for coexistence were met in 91% of draws for the constant temperature treatment, 97% for the low fluctuation treatment, and 99.9% for the high fluctuation treatment.

### Population simulations

**Fig. 2:**
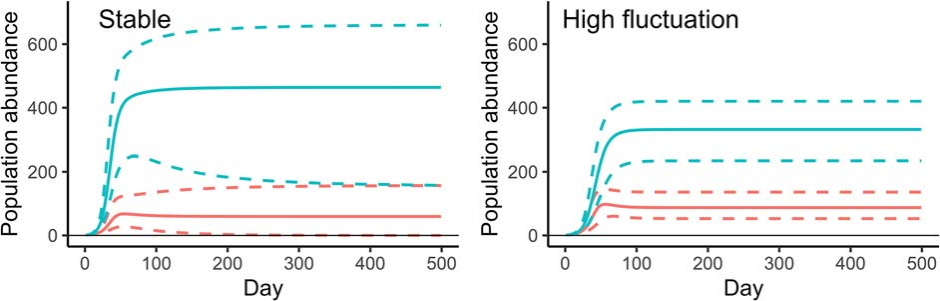
Simulated population dynamics of *D. magna* (red lines) and *D. pulex* (blue lines) under competition. For each graph, 100 000 simulations starting with one individual of each species were run, with population dynamic parameters being drawn from a normal distribution based on mean and SE of parameter estimates provided in Table 1. Solid lines represent median abundances, and dashed lines the 2.5 and 97.5% percentiles. Parameter estimates (for *D. pulex*) under low temperature fluctuation were intermediate between constant and high fluctuation, so only results applying the latter two are shown here.

The results from the population simulations suggest that the estimated species-specific intrinsic growth rates and competition coefficients frequently allow for coexistence between these two species. Qualitatively this result is independent of the level of temperature fluctuations (Fig. 2). However, the relative abundance of the two species under equilibrium is changed, with a reduced abundance of *D. pulex* (median abundance decreased by 29%) when simulating with parameter values from fluctuating conditions compared to those from constant conditions, and a simultaneous increase in *D. magna* (increase by 47%, Table 2). Moreover, whereas a considerable number of simulated *D. magna* populations went extinct in simulations based on constant conditions, this was negligible for fluctuating conditions (Table 2). To evaluate the sensitivity of this conclusion to model choice, we repeated the simulations with all pairwise combinations of models for *D. pulex* and *D. magna* that were within two AICc of the top model (see table S3 for parameter estimates of these). All these simulations showed the same qualitative result as when using the top models. Under high temperature fluctuations, equilibrium abundance of *D. magna* increased by 65 - 110% (depending on model pair) while abundance of *D. pulex* decreased by 32 - 39% compared to at a constant temperature (Table S2, Figs S3–S7).

**Table 2:** Median (95% CI) equilibrium population size and percentage of populations going extinct for *Daphnia magna* and *D. pulex* in competition simulations using the parameter estimates provided in Table 1. A total of 100 000 populations were simulated for 1000 days before obtaining population sizes.

Environment	*D. magna*		*D. pulex*	
	Median	% extinct	Median	% extinct
Constant	60 (0, 158)	5.13	464 (159, 662)	0.40
High fluctuation	88 (53, 136)	< 0.01	332 (234, 421)	< 0.01

## DISCUSSION

The current study provides an experimental demonstration of how temperature variation influences intra- and inter-specific competition coefficients in two zooplankton species, ultimately shaping their relative abundances and risks of extinction. Specifically, we show that under constant temperature conditions, one of the species (*D. magna*) was greatly outnumbered by their competitor (*D. pulex*). This competitive advantage was supported by population simulations that applied the estimated species-specific population dynamic parameters, where the median equilibrium population abundance of *D. pulex* was 7.7 times that of *D. magna*. Furthermore, when considering uncertainty in these parameter estimates during simulations, we observed a considerable possibility for extinction of the inferior competitor under constant conditions. In fact, calculations of ND and RFD showed that our estimated parameter distributions for constant conditions contained some parameter sets that precluded stable coexistence. Under fluctuating temperature conditions, the numerical dominance by *D. magna* was considerably reduced in the empirical data, and the median equilibrium population abundance of *D. pulex* was only 3.8 times that of *D. magna* in the high fluctuation scenario. Furthermore, the occurrence of extinction of the inferior competitor in the simulations became negligible under these conditions, and all parameter sets drawn from the estimated distributions met the requirements for stable coexistence.

Previous studies have examined the occurrence of and potential for coexistence between *D. magna* and *D. pulex*. Some of the early work suggested that the two species impose strong interspecific competition on each other due to low niche differentiation, and that *D. pulex* is the stronger competitor of the two (Hanski and Ranta, 1983; Bengtsson, 1986). Observations of co-occurrence patterns in rock pools on Baltic islands during a single year led Hanski and Ranta (1983) to conclude that the low niche differentiation prevents stable coexistence, and they invoked metapopulation dynamics as the mechanism that causes the two species to occasionally co-occur, with species continuously colonizing or recolonizing some rock pools and going extinct from others. Furthermore, in competition experiments conducted under controlled constant environmental conditions (including temperature), one of the species always went extinct (Bengtsson, 1986). However, when reared in outdoor mesocosms exposed to ambient conditions for a complete growth season, competition rarely resulted in extinctions (Bengtsson, 1986). More recently, Luo *et al.* (2022) parameterized a spatially explicit metacommunity model that builds on coexistence theory using a 35-year time series of three species of *Daphnia* (including *D. magna* and *D. pulex*) from 546 rock pools. Their results suggest that niche differences between these species were large relative to fitness differences. This resulted in competition effects on local extinction rates to be weak in these natural populations, with only a marginal improvement in model fit when including such an effect compared to models without competition.

Our results support the view that these species may coexist even in the absence of metapopulation dynamics. However, we also provide experimental evidence of how variation in temperature may increase the probability of coexistence. Specifically, best-fit models (Table 1) reveals that the competitively superior *D. pulex* experiences an increase in the intraspecific competition coefficient and a reduction in intrinsic population growth rate with increasing levels of temperature fluctuations. In comparison, the inferior *D. magna* appear less affected by levels of temperature fluctuations. The second most supported model for *D. magna* shows a similar pattern, with temperature fluctuations only influencing the intraspecific competition coefficient, and with the magnitude of this effect being relatively small (Table S3). This suggests that the likelihood of stable coexistence of these two species is improved under fluctuating temperature conditions, which is confirmed by our calculations of ND and RFD, in which the number of parameter sets precluding stable coexistence decreased as temperature became more variable. This mirrors the observations on coexistence in stable laboratory conditions vs. in outdoor mesocosms mentioned above (Bengtsson, 1986), and suggest that effects of environmental variation on coexistence may play a role also in natural populations of these two species. It should be noted, however, that the duration of the current experiment was limited, and that the documented positive effect of temperature fluctuations are not necessarily sufficient to ensure longer-term coexistence. Thus, it may well be the case that interspecific competition causes these two species to coexist less frequently in nature than expected based on a random distribution of species. However, what our results suggest is that the frequency of coexistence would be expected to be even lower in the absence of natural temperature fluctuations. Furthermore, although we were able to demonstrate a qualitative effect of environmental variation on the potential for species coexistence in a simple experimental setting, such effects may play out differently in natural populations. For example, in the current study we used a single clone of each species. In contrast, natural populations of daphnia typically consist of a variety of clones with different characteristics (Fossen *et al.,* 2018). It seems possible that this might influence the outcome of competitive interactions between the two species and how this might differ under different environmental conditions.

The mechanism behind the observed effect of temperature variation remains elusive. It appears reasonable to assume that the thermal performance curves of the two species that were used in the experiment differ to some extent, as such curves have been found to differ even among clones within one of the species (*D. magna*, Fossen *et al.,* 2018). Additional studies of the performance and dynamics of the individual species when experiencing temperature variation in isolation (i.e., in monocultures without interspecific competition) could help shed more light on this, and would also allow more precise estimates of intraspecific competition and intrinsic population growth. Effects of being exposed to different stable temperatures on the relative abundance of these two species when reared in competition has also been observed previously (Maszczyk *et al.,* 2024). Modelling work has indeed shown that such effects combined with short-term variation in temperature can promote species coexistence (Liu *et al.,* 2021), and the current work provides empirical support for this effect. Alternatively, more complex mechanisms involving effects of temperature regime on the bacterial community in combination with species-specific responses to this may have contributed. First, bacteria are a food source for daphnia (Wenzel *et al.,* 2021), and the effectiveness of feeding on these will depend on the mesh size of their filtering apparatus, which differs among species (Brendelberger and Geller, 1985). Furthermore, the bacteria community may influence the gut microbiome with corresponding effects on reproductive output (Obrestad *et al.,* 2022). Second, the feeding regime likely resulted in fluctuating algae concentrations, with the extent of these potentially differing among temperature regimes, and with the possibility that different species responded differently to this. Finally, the observed impacts of temperature variation might have occurred independently of any effect of competition if the two species differ in how they allocate resources to the competing demands of reproduction and somatic growth depending on temperature (Maszczyk *et al.,* 2024). Distinguishing between these different potential mechanisms was not possible in the current study. A more detailed understanding of the underlying processes would perhaps have been possible in the presence of data (e.g. life history traits, thermal performance curves) that could describe the two clones we used (Bruijning *et al.,* 2025), however such data were not available for the current study.

Although temperature variation reduced the dominance of *D. pulex* and increased the probability for coexistence, competitive exclusion was not the rule even under constant conditions. Similar apparent stable coexistence between these two species under stable environmental conditions was recently observed by Maszczyk *et al.* (2024). This suggests that some niche differentiation must be present even in the simplest of habitats (glass jars) where the potential for spatial niche partitioning appears absent. Our estimate of niche differentiation under constant conditions was 0.31, on a scale from 0 (identical niches) to 1 (no niche overlap). One possible explanation for the niche differentiation could be that the two species differ in their efficiency to utilize different components of the available food. The Shellfish Diet used in this study contains four different inactivated marine microalgae (*Isochrysis sp*., *Pavlova sp.*, *Tetraselmis sp.* and *Thalassiosira pseudonana*), which range in size between 4 and 20 micrometers. Although the mesh sizes of the filtering apparatus of *D. magna* and *D. pulex* differ slightly (Brendelberger and Geller, 1985), and these mesh sizes determine their food size selection (Gophen and Geller, 1984), it is not expected that the efficiency of ingesting particles within this size range differs between them (Nadin-Hurley and Duncan, 1976). However, as documented in previous studies in our laboratory, rearing under these conditions allows for growth of biofilm on the jar surfaces (Issa *et al.,* 2020), which can provide an additional food resource to the *Daphnia* populations (Siehoff *et al.,* 2009), and it is not known to what extent such utilization may differ between the two species. Furthermore, whereas sedimentation of phytoplankton makes them unavailable for *Daphnia* that filters the medium in the water column, *Daphnia* may also browse through the sediment to stir up particles that are subsequently ingested by filter feeding. The extent of such behaviour may differ among species, as it has been shown to vary among clones of *D. magna* (Arbore *et al.,* 2016). Finally, we have previously documented bacterial growth in the medium under our laboratory conditions (Obrestad *et al.,* 2022), and the ability to utilize these as a food source may also differ between the two species (Brendelberger, 1991).

## CONCLUSIONS

Our results provide empirical support for previous model results showing how short-term variation in temperature can promote species coexistence. Such improved understanding of how environmental fluctuations impact species coexistence is particularly important in the light of global climate change. As the climate warms, it is also becoming more erratic, with more frequent and intense extreme weather events (IPCC, 2022). Weather variability is also changing, but the direction and magnitude of these changes have proven difficult to predict and will likely differ among weather variables and regions (Lewis and King, 2017). Thus, coexistence of competing species could be both disrupted and promoted by these changes through effects on competitive interactions. Species distributions are also expected to undergo major shifts as the climate continues to change, bringing together new assemblages of species and rewiring ecological communities (Urban *et al.,* 2012; Wallingford *et al.,* 2020). As illustrated by the current work, predicting how natural systems are likely to change under different climate scenarios requires in-depth understanding of the mechanisms and conditions that influence species coexistence and the role of environmental variation in these processes.

## Supplementary Material

Supplementary_materials_fbag019

## Data Availability

The data used in this article are available in a supplementary file.

## Notes

Corresponding Editor: Lars-Anders Hansson
