# Supplementary material for: The effect of environmental variation on stable coexistence of competitors: experimental evidence from zooplankton (Daphnia magna and D. pulex)
Source: J Plankton Res. 2026 Mar 28;48(2):fbag019. doi: 10.1093/plankt/fbag019 (PMC13032039; doi:10.1093/plankt/fbag019)
Supplement: Supplementary_materials_fbag019 [file supplementary_materials_fbag019.zip › JPR Suppl.docx]

**Supporting information for:**

**The effect of environmental variation on stable coexistence of competitors: experimental evidence from zooplankton (*Daphnia magna* and *D. pulex*)**

Sigurd Einum^1*^, Tim Burton^1, 2^, Silje M. Larsen^1, 2^, Varsha Yadav^1^, Aline M. Lee^1, 3^

1. Centre for Biodiversity Dynamics, Department of Biology, Norwegian University of Science and Technology, Realfagbygget, NO-7491 Trondheim, Norway
2. Norwegian Institute for Nature Research, Høgskoleringen 9, 7034, Trondheim, Norway
3. Gjærevollsenteret, Norwegian University of Science and Technology, Realfagbygget, NO-7491 Trondheim, Norway

*corresponding author

Table S1. AICc comparison of population abundance models for *D. magna* and *D. pulex* when reared in competition. All models contributing to the cumulative weighted AICc are listed.

| Species | Treatment | Census nr. | Species x treatment | Species x census nr. | Block | AICc | Δ AICc | AICc wt | Cum wt |
| --- | --- | --- | --- | --- | --- | --- | --- | --- | --- |
| + | + | + | + | + | - | 6138.5 | 0.00 | 0.67 | 0.67 |
| + | + | + | + | + | + | 6140.7 | 2.18 | 0.23 | 0.90 |
| + | + | + | + | - | - | 6143.0 | 4.42 | 0.07 | 0.97 |
| + | + | + | + | - | + | 6145.1 | 6.54 | 0.03 | 1.00 |

Table S2. AICc comparison of population growth models for *D. magna* and *D. pulex* when reared in competition. Different versions of the model were fitted, either including or excluding (indicated by + or -, respectively) temperature treatment (trt) effects on the intraspecific competition coefficient (*a_ii_* or *a_jj_*), interspecific competition coefficient *(a_ij_* or *a_ji_*) and *r* (intrinsic rate of increase). All models contributing to the cumulative weighted AICc are listed.

|  | Model | Intrasp~trt | Intersp~trt | r~trt | AICc | ΔAIC | weight | Cum wt |
| --- | --- | --- | --- | --- | --- | --- | --- | --- |
| *D. magna* | 1 | - | - | - | -390.84 | 0.00 | 0.34 | 0.34 |
|  | 2 | + | - | - | -390.34 | 0.50 | 0.27 | 0.61 |
|  | 3 | - | + | - | -388.68 | 2.16 | 0.12 | 0.72 |
|  | 4 | + | + | - | -388.14 | 2.70 | 0.09 | 0.81 |
|  | 5 | - | - | + | -387.86 | 2.98 | 0.08 | 0.89 |
|  | 6 | + | - | + | -387.68 | 3.16 | 0.07 | 0.96 |
|  | 7 | - | + | + | -385.51 | 5.33 | 0.02 | 0.98 |
|  | 8 | + | + | + | -384.97 | 5.87 | 0.02 | 1.00 |
| *D. pulex* | 1 | + | + | + | -833.08 | 0.00 | 0.47 | 0.47 |
|  | 2 | + | - | + | -831.43 | 1.65 | 0.20 | 0.67 |
|  | 3 | + | - | - | -831.17 | 1.90 | 0.18 | 0.85 |
|  | 4 | + | + | - | -830.74 | 2.33 | 0.15 | 1.00 |

Table S3. Parameter estimates ± SE of models with ΔAICc < 2 additional to those given in Table 1. Single estimates are given for parameters that did not depend on the environment (Common) or separately for each environment for parameters that depended on the environment. Model numbers refer to Table S2.

|  | Common | Constant | Low fluctuation | High fluctuation |
| --- | --- | --- | --- | --- |
| *D. magna*, model 2 |  |  |  |  |
| *a_ii_* |  | 0.00801 ± 0.00136 | 0.00568 ± 0.00086 | 0.00683 ± 0.00115 |
| *a_ij_* | 0.00130 ± 0.00020 |  |  |  |
| *r* | 0.11783 ± 0.01434 |  |  |  |
| *c* | 1.57762 ± 0.30768 |  |  |  |
| *D. pulex*, model 2 |  |  |  |  |
| *a_jj_* |  | 0.00182 ± 0.00011 | 0.00176 ± 0.00016 | 0.00254 ± 0.00019 |
| *a_ji_* | 0.00216 ± 0.00046 |  |  |  |
| *r* |  | 0.18123 ± 0.01276 | 0.16486 ± 0.01200 | 0.14791 ± 0.01213 |
| *c* | 0.86371 ± 0.10094 |  |  |  |
| *D. pulex*, model 3 |  |  |  |  |
| *a_i_* |  | 0.00178 ± 0.00012 | 0.00176 ± 0.00016 | 0.00262 ± 0.00018 |
| *a_j_* | 0.00200 ± 0.00047 |  |  |  |
| *r* | 0.16630 ± 0.00880 |  |  |  |
| *c* | 0.80953 ± 0.09630 |  |  |  |

Table S4. Median (95% CI) of equilibrium population size of *D. pulex* and *D. magna* in competition based on simulations using the parameter estimates from all models with ΔAICc < 2 in Table 1. 10000 populations of each species were simulated for 1000 days before obtaining population sizes. Model numbers refer to Table S2.

| Models used | Environment | *D. magna* | *D. pulex* |
| --- | --- | --- | --- |
| magna model 1, pulex model 2 | Constant | 55 (2, 107) | 485 (382, 593) |
|  | High fluctuation | 91 (54, 141) | 316 (237, 399) |
| magna model 1, pulex model 3 | Constant | 50 (0, 102) | 506 (402, 627) |
|  | High fluctuation | 92 (57, 138) | 311 (239, 385) |
| magna model 2, pulex model 1 | Constant | 44 (0, 115) | 494 (286, 658) |
|  | High fluctuation | 83 (49, 141) | 334 (240, 420) |
| magna model 2, pulex model 2 | Constant | 44 (6, 90) | 497 (408, 594) |
|  | High fluctuation | 85 (50, 149) | 321 (237, 401) |
| magna model 2, pulex model 3 | Constant | 41 (2, 87) | 515 (420, 625) |
|  | High fluctuation | 86 (53, 147) | 314 (236, 387) |

**
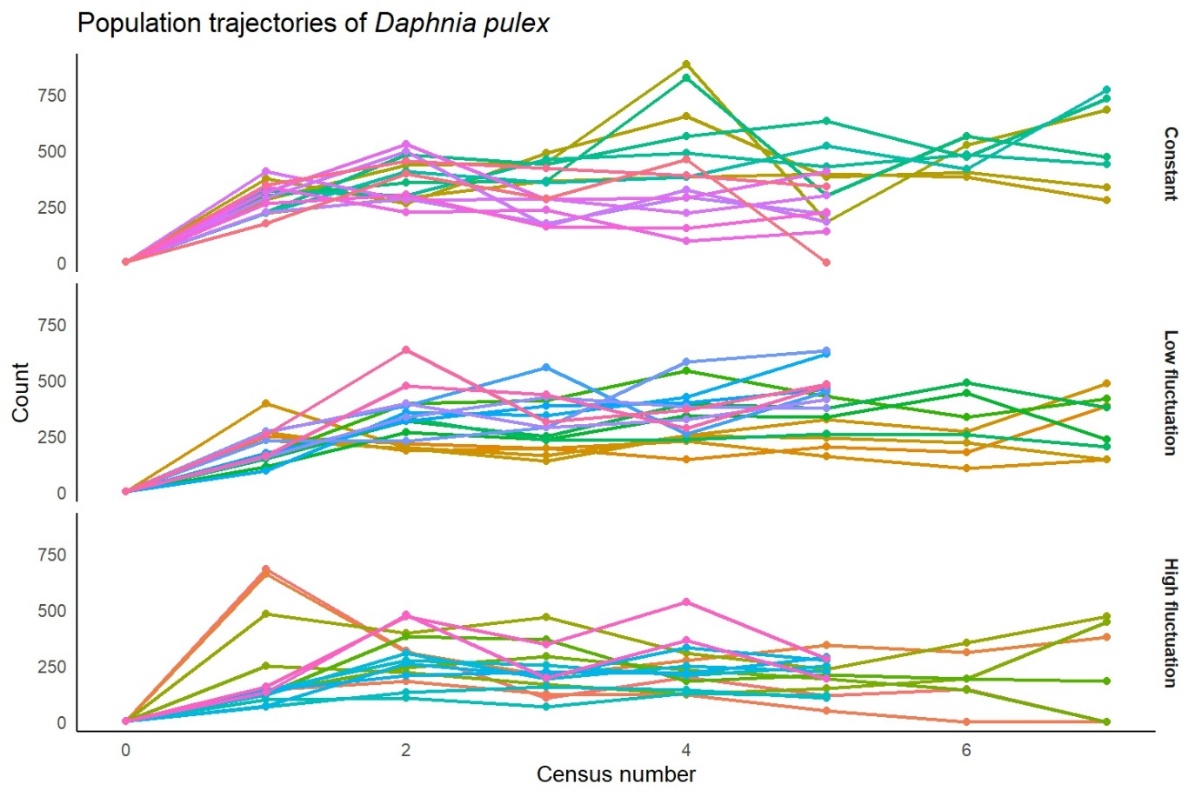
**

**
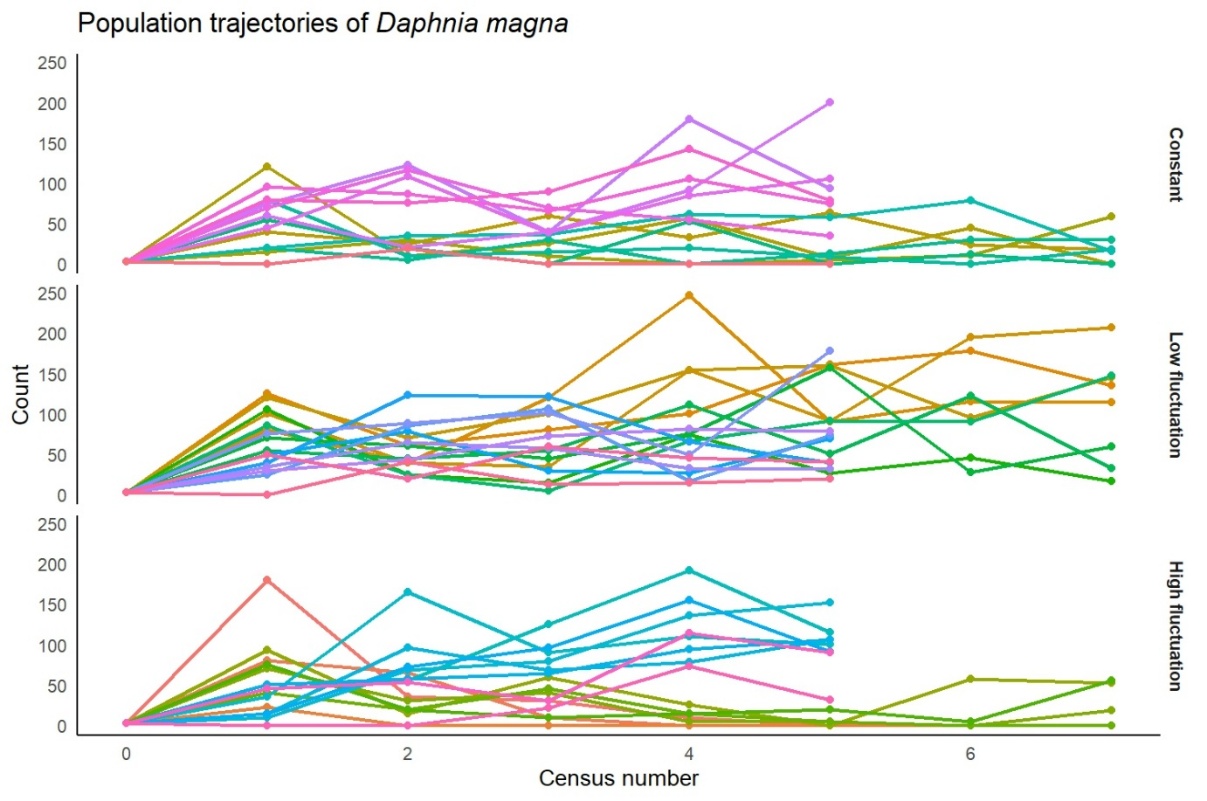
**

Fig. S1. Population abundance of *Daphnia pulex* and *D. magna* over time when in competition under the three temperature regimes; constant, low fluctuations, and high fluctuations (HF). Different coloured lines represent different population replicates. Experiments were run in two blocks, with populations from the first block having seven censuses, and with populations from the second block having five censuses.


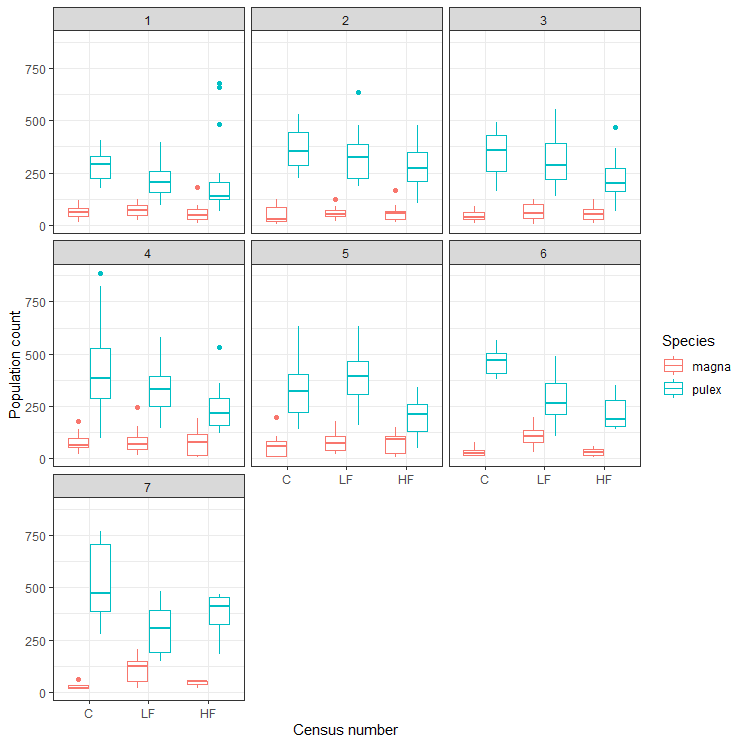


Fig. S2. Population abundance of *Daphnia magna* and *D. pulex* when in competition under the three temperature regimes; constant (C), low fluctuations (LF) and high fluctuations (HF). Separate panels are given for censuses spaced one week apart.


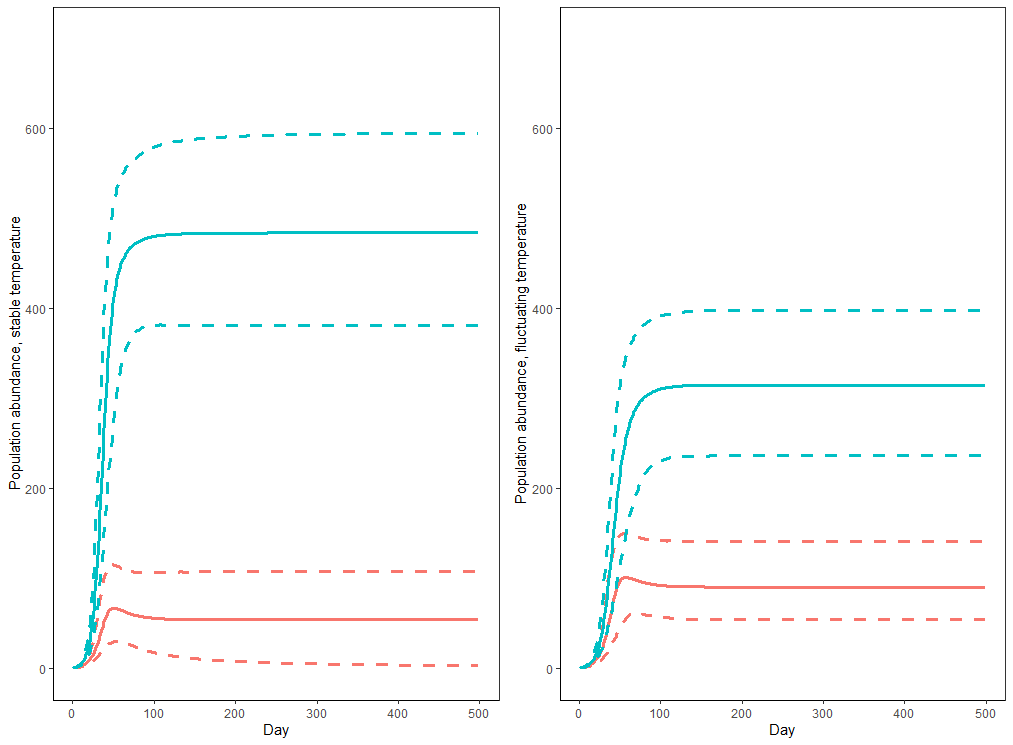


Fig. S3. Simulated population dynamics of *D. magna* (red lines) and *D. pulex* (blue lines) under competition when applying model 1 for *D. magna* (Table 1) and model 2 for *D. pulex* (Table S3). Other details are as in Fig. 2.


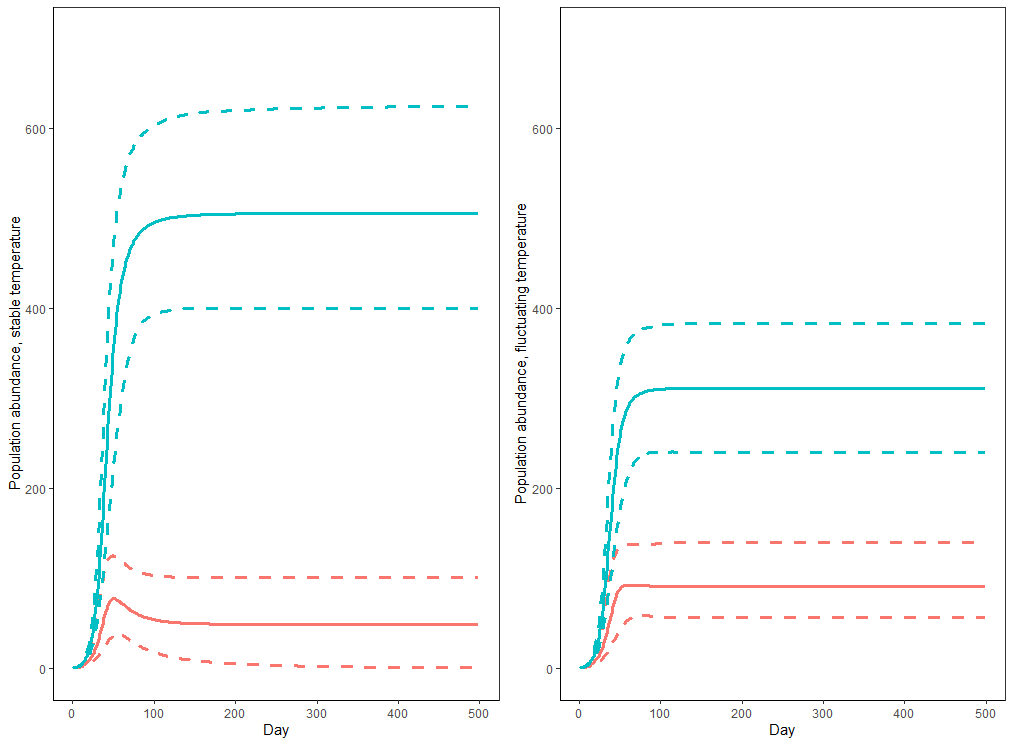


Fig. S4. Simulated population dynamics of *D. magna* (red lines) and *D. pulex* (blue lines) under competition when applying model 1 for *D. magna* (Table 1) and model 3 for *D. pulex* (Table S3). Other details are as in Fig. 2.


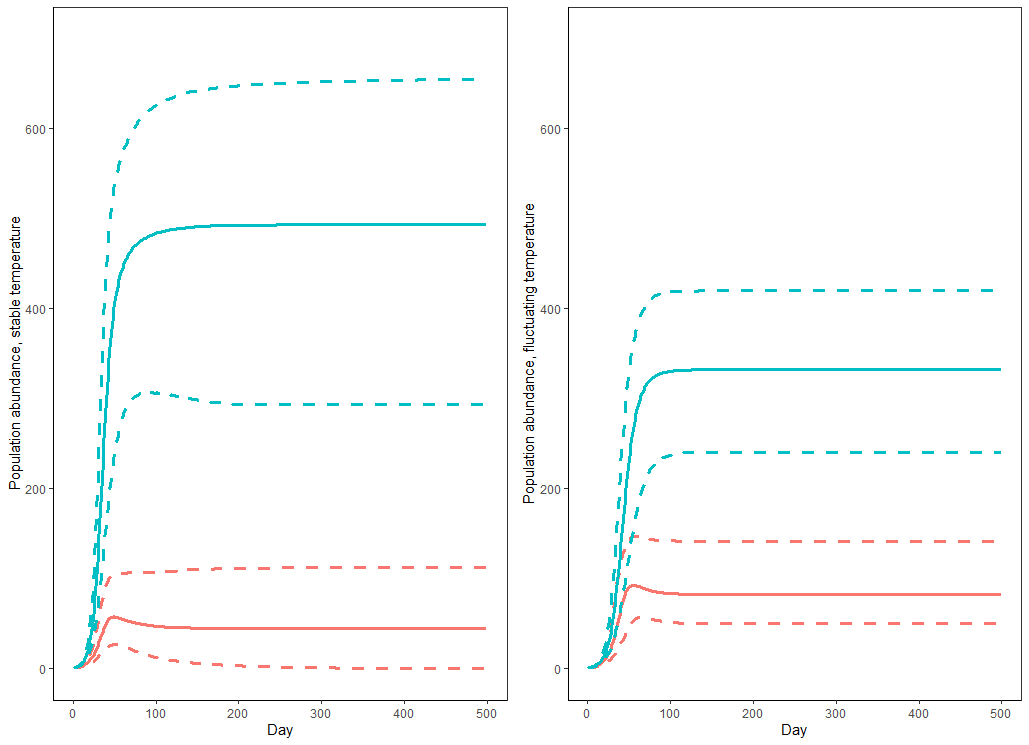


Fig. S5. Simulated population dynamics of *D. magna* (red lines) and *D. pulex* (blue lines) under competition when applying model 2 for *D. magna* (Table S3) and model 1 for *D. pulex* (Table 1). Other details are as in Fig. 2.


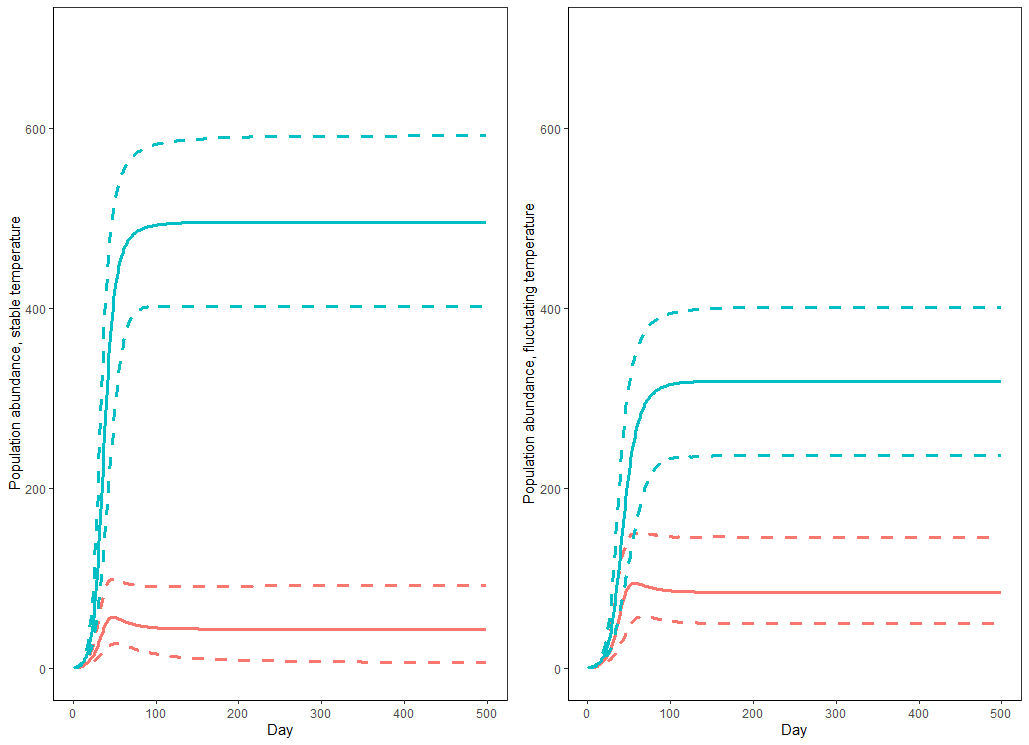


Fig. S6. Simulated population dynamics of *D. magna* (red lines) and *D. pulex* (blue lines) under competition when applying model 2 for *D. magna* and model 2 for *D. pulex* (Table S3). Other details are as in Fig. 2.


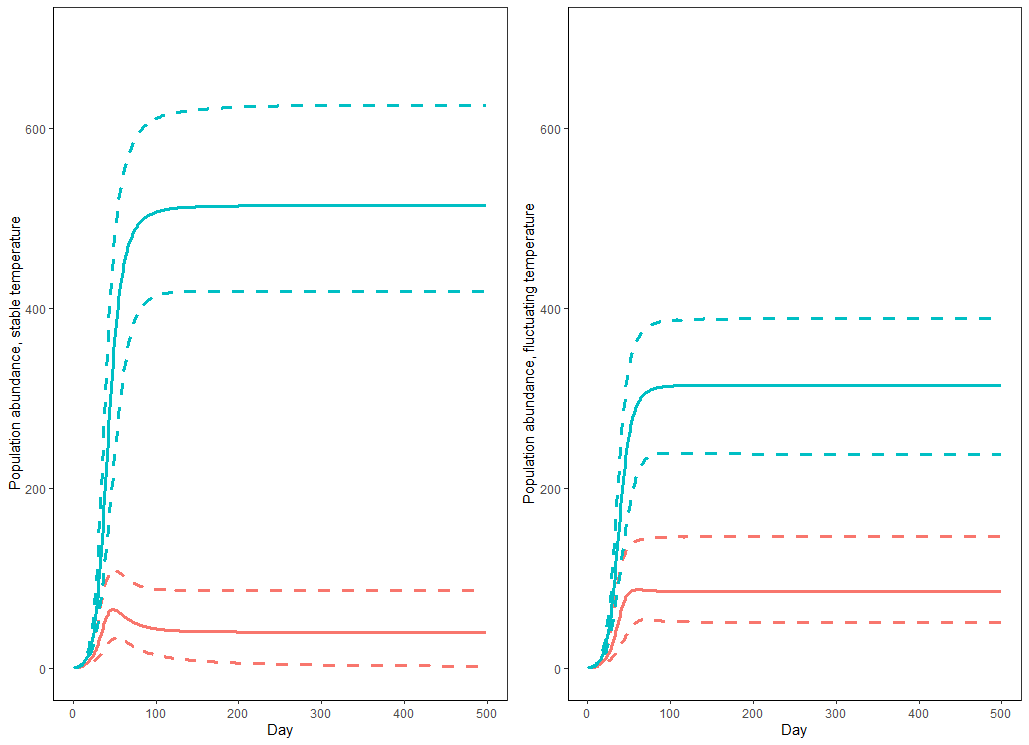


Fig. S7. Simulated population dynamics of *D. magna* (red lines) and *D. pulex* (blue lines) under competition when applying model 2 for *D. magna* and model 3 for *D. pulex* (Table S3). Other details are as in Fig. 2.
